# The ‘French exception’: the right to continuous deep sedation at the end of life

**DOI:** 10.1136/medethics-2017-104484

**Published:** 2017-10-22

**Authors:** Ruth Horn

**Affiliations:** Oxford Big Data Institute, Wellcome Centre for Ethics and Humanities, Oxford, UK

**Keywords:** end-of-life, law, pain management, euthanasia

## Abstract

In 2016, a law came into force in France granting terminally ill patients the right to continuous deep sedation (CDS) until death. This right was proposed as an alternative to euthanasia and presented as the ‘French response’ to problems at the end of life. The law draws a distinction between CDS and euthanasia and other forms of sympton control at the end of life. France is the first country in the world to legislate on CDS. This short report describes the particular context and underlying social values that led to this piece of legislation, and explores its meaning in the wider French context.

In February 2016, a law was adopted in France granting terminally ill patients who refuse life-sustaining treatment (LST) the right to continuous deep sedation (CDS).^1^ The right to CDS was presented as the ‘French exception’ or the ‘French response’ to euthanasia. The expression ‘French exception’ is used in France to distinguish the country from other countries with regard to the relationship between the State and its social systems such as, culture, language or, as in this case, the healthcare system.^2^ The French republican model provides that the State regulates these systems in order to preserve society’s common values, or the ‘general will’, rather than individual interests as is the case in more liberal societies.^3^ What social values does the State seek to preserve with the ‘right to CDS’? In the following, I will describe the socio-legal context that gave rise to this law, and discuss the meaning of the law in the French context.

Since the late 1970s, the French end-of-life debate has been characterised by an asymmetry between physicians’ and patient rights. Although the paternalistic approach has been criticised in the public debate and several laws on patient rights have been adopted, respect for patient autonomy has struggled to find a place in French medical practice. A first attempt to strengthen patient rights was made in 2002 when a law was adopted that acknowledged, among others, the right to refuse treatment.^4^ However, in 2003, the case of Vincent Humbert, a paraplegic patient who claimed his right to die, highlighted the uncertainties of physicians regarding the legality of withdrawing LST, such as clinically assisted hydration and nutrition.^5^ This case attracted much media attention and generated an important parliamentary report in 2004.^6^ This report led to the adoption of the law ‘on patient rights and the end of life’ in 2005. Yet, the law did not, as the name could suggest, create a framework to enhance patient rights. Rather, it reassured physicians about the legality to withdraw LST if they deem it necessary.^5 7 8^ Even though the law stipulated that patients ‘can refuse every treatment’, physicians were not required to accept their refusal. Rather the law stated that the physician should ‘do all that is possible to convince the patient to continue the treatment when the refusal endangers the latter’s life’. Similarly, in case of incompetent patients, advance decisions to refuse treatment only had advisory value but were not legally binding.

Since the 2005 law came into force, a series of parliamentary reports have evaluated its relevance and highlighted persisting problems with regard to: pain control, respect for patient autonomy and decisions to withdraw LSTs.^9–12^ Cases widely covered in the media, such as that of Hervé Pierra^13^ or Vincent Lambert,^14^ confirmed the need for further legal clarification. The first case revealed deficiencies in adequate pain management of a terminally ill patient who, following the withdrawal of his feeding tube, subsequently suffered from heavy seizures for over 6 days. The second case involves a patient in a minimally conscious state who has been kept alive since 2008 due to protracted litigation engaged by his parents. The latter opposed to the withdrawal of LSTs initiated by the medical team in 2012 and, according to his wife, in agreement with the patient’s precedent wishes. In 2015, the Lambert was taken to the European Court of Human Rights which decided in favour of treatment withdrawal. The decision was backed by the Conseil d’Etat in 2017.^14^ Both cases demonstrate the absence of clear decisional procedures regarding the respect for patients' past beliefs and wishes that, at least until 2017, has characterised the legal landscape in France.

In 2015, in an attempt to address the insufficiencies of the law, the parliamentarians Alain Claeys and Jean Leonetti submitted a bill on ‘new rights for patients and terminally ill persons’.^15^ This bill sought to ensure that patients can refuse LST ‘without having to suffer’ by giving them the right to  CDS until death. France thus became the first country to consider a right to CDS. According to international guidelines, this extreme form of sedation, which renders patients unconscious until death, should be used only under strict monitoring and as a last resort option to manage intractable terminal suffering.^16^ Claeys and Leonetti’s proposition highlighted the concern that physicians could refuse to manage patients’ symptoms when LST is withdrawn. For many years, in France, the use of strong painkillers at the end of life was associated with euthanasia. As a result of this, many physicians were reluctant to use sedation and opiates at the end of life which in turn led to an extreme fear of terminal suffering among patients, healthcare professionals and the public.^5^ Yet, paradoxically, this fear also led some physicians to use lethal cocktails of analgesics with the intention to hasten patients’ death in order to efficiently terminate suffering.^8^ Consequently, when the 2015 Bill was submitted for a first reading to the National Assembly, a large majority of parliamentarians saluted this proposition as a way to fulfil president François Hollande’s electoral promise of 2012 to introduce a law allowing the terminally ill ‘to benefit from medical assistance to end their lives with dignity’.^17^

Following further discussions, an amended version of the bill was adopted by the Senate and entered into force on 3 February 2016. In the final version of the proposal, the right to CDS for any patient refusing LST has been limited to patients for whom death is imminent (up to 2 weeks) and pain is refractory (unmanageable by any other means). In addition, in cases where the terminally ill patient has lost capacity to express his/her will, the physician is now required to provide CDS when withdrawing LST.

Whether the law may be regarded as progressive in terms of assisted dying or criticised as opening the back door to euthanasia, the proposed ‘French exception’ could be seen as first and foremost an expression of a lack of trust in French physicians to effectively control pain at the end of life. The need to grant citizens a right to request the most extreme option of pain management is the result of doctors failing to fulfil their duty to sufficiently address patients’ pain and suffering. Some argue that the French decision to legislate on CDS, but not on euthanasia, marks the moral difference drawn between both practices.^18^ The French law makes CDS a ‘sui generis end-of-life practice’ that can be requested by a patient under certain conditions.^19^ CDS becomes herewith more than a medical response to severe pain. Moreover, by making it a requirement to provide CSD in every case where LST is withdrawn, CDS loses its purpose of controlling only refractory symptoms. As Raus *et al* 18 state, there is no justification for always accompanying withdrawal of LST by CDS. The potential disproportionality of providing CDS where there is no medical need for such an important intervention with possible, although disputable, life-shortening effect,^20 21^ raises ethical questions. It blurs the boundaries between CDS as a means to respond to refractory pain and CDS as a means to hasten death.^22^ Holm argues that CDS accompanied by the withdrawal of LST is very similar to euthanasia.^23^ Where this combination of practices is standard procedure, CDS needs to be carefully scrutinised to make it distinct from euthanasia, if this is what the French law intends.

In the French context, the blur of moral boundaries could result in two scenarios: (1) it could increase uncertainties and thus the reluctance of French physicians to use CDS, and therefore, physicians could avoid accepting patients’ wishes to withdraw LST; (2) some physicians could use CDS as a means of performing euthanasia (depending on the amount of sedatives administered) but without being monitored, as is normally the case in countries that have adopted legislation on euthanasia.^24^ The former is particularly problematic in the French context which is characterised by physicians’ attachment to provide treatment at the end of life even where this is futile.^5 8^ Given these possible scenarios, it can be argued that rather than granting terminally ill patients the right to request CDS and requiring doctors to accompany the withdrawal of LST for incompetent patients by CDS, French legislation should have aimed for improved pain management training and supporting guidelines on how and when to use CDS.
